# International regulatory landscape and integration of corrective genome editing into in vitro fertilization

**DOI:** 10.1186/1477-7827-12-108

**Published:** 2014-11-24

**Authors:** Motoko Araki, Tetsuya Ishii

**Affiliations:** Office of Health and Safety, Hokkaido University, Sapporo, 060-0808 Japan

**Keywords:** Genome editing, ZFN, TALEN, CRISPR/Cas, Embryonic stem cells, Zygote, Embryo, Assisted reproductive technology, In vitro fertilization, Genetic disease, Prevention, Germline gene modification, Regulations

## Abstract

Genome editing technology, including zinc finger nucleases (ZFNs), transcription activator-like effector nucleases (TALENs), and clustered regularly interspaced short palindromic repeat (CRISPR)/Cas, has enabled far more efficient genetic engineering even in non-human primates. This biotechnology is more likely to develop into medicine for preventing a genetic disease if corrective genome editing is integrated into assisted reproductive technology, represented by *in vitro* fertilization. Although rapid advances in genome editing are expected to make germline gene correction feasible in a clinical setting, there are many issues that still need to be addressed before this could occur. We herein examine current status of genome editing in mammalian embryonic stem cells and zygotes and discuss potential issues in the international regulatory landscape regarding human germline gene modification. Moreover, we address some ethical and social issues that would be raised when each country considers whether genome editing-mediated germline gene correction for preventive medicine should be permitted.

**Electronic supplementary material:**

The online version of this article (doi:10.1186/1477-7827-12-108) contains supplementary material, which is available to authorized users.

## Background

Germline (oocyte, sperm, zygote, and embryo) gene modification has been considered to be efficacious against some genetic diseases due to its impact on the entire body of the offspring. However, there has emerged a global consensus that such gene modifications should be forbidden owing to safety concerns [1–4], unprecedented informed consent [1, 2], challenges to human dignity [5], and the potential for permanent negative impact on future generations, including its abuse for eugenics or enhancement (the parental pursuit of specific traits for non-medical reasons) [2, 3, 6–8]. Indeed, human germline gene modification is largely forbidden by law or guidelines even in countries that are permissive to human embryonic stem cell research [9].

Meanwhile, in the late 90′s, the infusion of oocyte-cytoplasm, including mitochondria, was conducted to enhance the viability of oocytes in the USA [10]. This ooplasmic transfer needs an oocyte donor and is a form of germline gene modification because it causes heteroplasmy in the resulting oocyte [11, 12]. Although ooplasmic transfer led to more than 30 childbirths, the Food and Drug Administration (FDA) decided to regulate this procedure owing to potential health risk to progeny [13]. More recently, mitochondrial replacement, including pronuclear transfer between embryos [14] and maternal spindle transfer between oocytes [15, 16], has been developed and is proposed as novel medicine in order to prevent maternal transmission of serious mitochondrial diseases that result from aberrant mitochondrial DNA (mtDNA) in patient’s oocyte. Mitochondrial replacement is also a form of germline gene modification because this procedure involves altering the mtDNA content of human oocytes or embryos. In addition, mitochondrial replacement as well as ooplasmic transfer require at the very least oocyte donation which could potentially cause ovarian hyperstimulation syndrome in female donors [9]. Currently, the US FDA weighs the merits of the mitochondrial replacement [17]. Moreover, the UK Department of Health (DH) considers lifting the ban of mitochondrial replacement that is illegal in the UK at present [18]. In response to the result of public consultation, the DH will consider the timing of the regulations to permit mitochondrial replacement [19]. Such possible regulatory changes, which occur in a few, but major countries, may impact the international regulatory landscape that prohibits human germline gene modification.

Recent advances in genetic engineering are also likely to impact the international regulatory landscape. Present-day genome editing technology, such as zinc finger nucleases (ZFNs), transcription activator-like effector nucleases (TALENs), and clustered regularly interspaced short palindromic repeat (CRISPR)/Cas system, have achieved far more efficient genetic engineering in higher organisms than the older techniques [20–23]. Of genome editing technologies, the application of ZFNs has already reached to a clinical stage in AIDS therapy that is based on the administration of human chemokine receptor 5(CCR5)-modified T cells [24]. ZFN-mediated homology-directed repair has achieved correction of the mutations associated with X-linked severe combined immune deficiency (SCID) and haemostasis in somatic cells [25, 26]. Moreover, gene corrections by ZFNs have been reported regarding induced pluripotent stem cells derived somatic cells biopsied from patients with sickle cell disease, alpha1-antitrypsin deficiency, and Parkinson’s disease [27–30]. With regard to CRISPR/Cas9, it was demonstrated that those engineered nucleases can correct a mutation in intestinal stem cells derived from patients with cystic fibrosis [31]. Specific elimination of mutant mitochondrial genomes in patient-derived cells was attained by a new form of TALENs that can localize to mitochondria and cleave different classes of pathogenic mtDNA mutations [32].

Most notably, two reports emerged in 2014, demonstrating that the microinjection of Cas9 or TALENs into one-cell-stage embryos led to efficient generation of targeted gene-modified non-human primates (NHPs) [33, 34]. In addition, the microinjection of Cas9 system into mouse zygotes successfully corrected a 1 bp deletion in a targeted gene and prevented the onset of cataracts in that mouse’s offspring [35]. Thereafter, some experts, including a Nobel laureate, suggest increasing feasibility of human germline gene modification mediated by genome editing [36–38]. In the genome editing of mammals, targeted gene modification is frequently carried out by simply microinjecting of genome editing system which consists of the nuclease mRNAs (or plasmids harboring the nuclease gene), single guide RNAs (sgRNAs for Cas9), and a homology-containing donor DNA template (if necessary) into animal embryos made by *in vitro* fertilization (IVF) or intracytoplasmic sperm injection (ICSI) [33–35, 39–48]. Remarkably, this microinjection process resembles ICSI, one of assisted reproductive technology (ART) to facilitate fertilization in fertility clinics. Mammalian embryonic stem cells (ESCs), including human ESCs, have also been more efficiently modified by genome editing [35, 42, 43, 49–53]. Thus, rapid advances in genetic engineering render germline gene modification more feasible in higher animals. Genome editing technology is more likely to develop into medicine for preventing a genetic disease if corrective genome editing is integrated into assisted reproductive technology (ART), including IVF and ICSI. Importantly, germline gene correction by genome editing does not require cell donation such as oocyte donation that is needed for ooplasmic transfer and mitochondrial replacement. However, there are many issues that still need to be addressed before genome editing-mediated germline gene correction for preventive medicine could occur. We herein investigated current status of genome editing which modifies mammalian zygotes and embryonic stem cells as well as international regulations with regard to human germline gene modification. As a consequence, it was predicted that there would occur regulatory issues surrounding genome editing-mediated germline gene correction worldwide when the efficiency of genome editing technology is further improved. Moreover, we discuss forthcoming ethical and social issues that corrective genome editing would raise in the field of reproductive medicine.

### Potential subjects for genome editing-mediated germline gene correction

**Figure 1:**
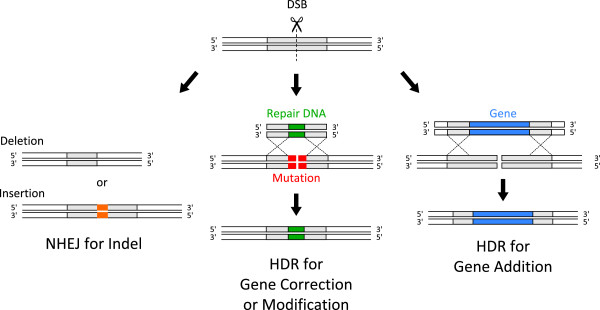
**Engineered nuclease-induced genome editing pathways.** Double-stranded breaks (DSBs) are induced at a targeted sequence by a microorganism-originated, engineered nuclease. Non-homologous end-joining (NHEJ) is a DSB repair pathway that ligates or joins two broken ends together, resulting in the introduction of small insertions or deletions (indels) at the site of the DSB. Homology-directed repair (HDR) is a DNA template-dependent pathway for DSB repair, using a homology-containing donor template along with a site-specific nuclease, enabling the insertion of single or multiple transgenes (gene addition) in addition to single-nucleotide substitutions in which an amino acid substitution of a protein occurs (gene modification), or a mutation is completely repaired in the resultant organism genome (gene correction).

Genetic engineering can produce site-specific mutations in cells or an organism. However, conventional genetic engineering can be extremely laborious and require time-consuming screens to identify a desired gene modification particularly in higher organisms. Genome editing technology is more efficient genetic engineering that can directly modify a gene within a genome in various organisms. This efficient gene modification is attained by a microorganism-originated, engineered nuclease that causes double-stranded breaks (DSBs) at a targeted sequence and induces DNA repair through non-homologous end-joining (NHEJ) or homology-directed repair (HDR) (Figure 1). The NHEJ is a DSB repair pathway that ligates or joins two broken ends together without a homologous template for repair, thus leading to the introduction of small insertions or deletions (indels) at the site of the DSB. The HDR is a DNA template-dependent pathway for DSB repair, using a homology-containing donor template along with a site-specific genome editing nuclease, enabling the insertion of single or multiple transgenes (gene addition) in addition to single-nucleotide substitutions in which an amino acid substitution of a protein occurs (gene modification), or a mutation is completely repaired in the resultant organism genome (gene correction). Remarkably, genome editing technologies do not leave marked genetic vestiges such as residual *loxP* sites that result from the Cre/loxP recombination system in transgenic mice, following the modifications. However, there are still some technical issues in genome editing. Identifying desired cells or animals which have an intentional mutation among arising variants still require careful screening, despite less laborious than conventional methods. Moreover, genome editing technology may fail to induce a biallelic modification in an animal, thereby resulting in only an animal with a monoallelic modification. The engineered nucleases could also cause off-target mutations other than desired gene modification in a target sequence [35, 39–41, 44, 49–51]. Furthermore, the microinjection of the nuclease mRNAs into zygotes may induce not only germline modifications but also mosaic modifications in which wild-type cells, including germline cells, and genetically modified cells coexist in the resultant animals [41, 47]. Therefore, the entire process of genome editing must be cautiously controlled by genetic analysis, meticulous screening, and sufficient characterization of resulting animals.

If corrective genome editing is integrated into ART, represented by IVF and ICSI, the major medical implications of the germline gene correction are preventive medicine rather than therapy, because this type of medical procedure aims at not the treatment of existing patients, but the prevention of transmission of a genetic disease to offspring [54]. For this reason, potential subjects would include those with congenital anomalies that are caused by chromosomal, monogenic, multifactorial, or environmental/teratogenic factors [55]. Among these, a monogenic disease would be the initial candidate for clinical application, since genome editing can efficiently repair such a small mutation in the human germline. However, medical use of genome editing for preventing transmission of a monogenic disease should be limited to cases where the medical benefits exceed the potential health risks associated with the genetic intervention, implying definite inheritance by the offspring. For instance, an autosomal recessive disease in which both parents are homozygous (e.g. cystic fibrosis [56], phenylketonuria [57]) or an autosomal dominant disease where at least one parent is homozygous (e.g. Huntington’s disease [58], familial adenomatous polyposis [59]) is likely to be considered. Genome editing-mediated germline gene correction which could potentially cause off-target mutations is not likely to be considered elsewhere for the time being because preimplantation genetic diagnosis (PGD) may circumvent an affected pregnancy by selecting IVF embryos with no suspected mutations [60].

Although clinical cases in which genome editing-mediated germline gene correction is efficacious and applicable might be confined to parents with a specific genetic background, as well as a monogenic disease, such cases will likely be found. If affected parents use a medical procedure that prevents offspring from inheriting their devastating disease, the public would sympathize with them, similar to the case in mitochondrial replacement [9]. One might assert that affected parents should not use such a risky genetic intervention, and should instead use donor gametes or donor embryos. However, the parents should not be forced to use these reproductive options. Most parents wish to have their own genetically-related healthy child. Therefore, the use of such a medical procedure could represent an alternative reproductive option.

### Approaches for genome editing-mediated gene correction

**Figure 2:**
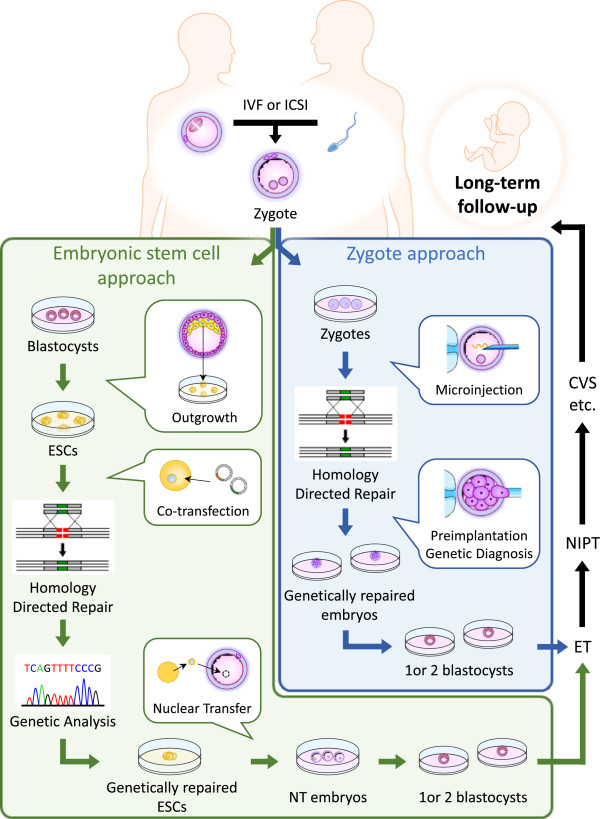
**Embryonic stem cell approach and zygote approach for genome editing-mediated gene correction to prevent a genetic disease.** Zygotes with a mutation are treated with genome editing-mediated gene correction via embryonic stem cell approach or zygote approach. After embryo screening by preimplantation genetic diagnosis, one or more embryos which have a corrected gene with no off-target mutations are subjected to embryo transfer. NIPT can be used to confirm the genetic condition of the fetus. Subsequently, CVS or amniocentesis can confirm whether a fetus has genetic mosaic mutations. Long-term follow-up is required even after a successful birth owing to the contribution of the modified germline to the entire body. CVS: chorionic villus sampling, ESCs: embryonic stem cells, ET: embryo transfer, ICSI: intracytoplasmic sperm injection, IVF: in vitro fertilization, NIPT: non-invasive prenatal genetic testing, NT: nuclear transfer.

We considered two possible approaches for genome editing-mediated germline gene correction to prevent definite inheritance of a genetic disease (Figure 2). If one attempts to repair a mutation directly in oocytes or embryos by means of an older homologous recombination technique, this attempt is likely to be a failure owing to its low efficiency. Therefore, genome editing-mediated gene correction in ESCs which are derived from a parent’s embryo made by IVF or ICSI could represent an alternative approach. Taking advantage of the self-renewal of ESCs, *in vitro* expansion and cryopreservation of ESCs enables repeated attempts to correct a mutation in a specific gene by genome editing. According to reports regarding genome editing of mammalian ESCs, the efficiencies of indel and gene addition are 14 to 91% by Cas9 and 0 to 83.49% by ZFNs or TALENs, respectively (Table 1). Importantly, the efficiency, 83.49% was achieved in a human ESC experiment for gene addition by ZFNs. The efficiency of NHEJ-mediated indel is high even in the simultaneous mutations of three loci (14%, 21%). In contrast, the efficiency of the HDR-mediated gene addition may depend on the selection of a targeted gene. Of note, both ZFNs and TALENs could not attain homozygous gene addition in the OCT4 of human ESCs [50, 51]. Compared with these results regarding indel and gene addition, the efficiency of targeted gene correction by ZFNs or Cas9 is low in human ESCs (Table 1). On a closer examination, however, the efficiency of gene correction of integrated GFP with ZFNs was 0.24%, a > 2400-fold increase as compared with gene correction without ZFNs in Human ESCs [52]. In mouse ESCs, the best result of gene correction, 44.4% was obtained in a case of a well-designed guide RNA in Cas9 system (Table 1), although two occurrences of off-target mutations were observed [35]. Off-target mutations were also observed in the three reports regarding gene addition in human ESCs [49–51]. In preclinical research, meticulously designing and validating Zinc finger domains in ZFNs, TALE subunits in TALENs, and sgRNAs in CRISPER/Cas which are specific to a target site of a gene is required in order to maximize the efficiency of desired gene modification and minimize the possibility of off-target mutations in genome editing-mediated gene correction.

**Table 1:** **Genome editing of mammalian embryonic stem cells**

Subject	Targeted Gene	Efficiency of Modification ^*^	Off-target Mutation	Genome Editing	Delivery Method	Ref.
**NHEJ for InDel**						
Rat haploid ESCs	Tet1, Tet2, Tet3	91% (Single locus), 45% (Double), 14% (Triple)	N.D.	Cas9	Plasmid/sgRNA	[53]
Mouse ESCs	Tet1, Tet2, Tet3, Sry, Uty	56 ~ 77% (Single; Tet1,2,3), 21% (Triple; Tet1,2,3)	No	Cas9	Plasmid	[43]
**HDR for Gene Addition**						
Human ESCs	GFP into CCR5	5.3%	Yes	ZFNs	Lentivirus	[49]
Human ESCs	PGK-Hygro^R^ into, PIG-A	0.19 ~ 83.49%	N.D.	ZFNs	Plasmid	[52]
Human ESCs	GFP (or PURO) into, AAVS1, OCT4, PITX	2.9 ~ 15.2% (AAVS1), 0% (OCT4, PITX3)	No (AAVS1, OCT4), Yes (PITX3)	ZFNs	Plasmid	[50]
Human ESCs	GFP (and/or PURO), into. AAVS1, OCT4, PITX3	9 ~ 22% (AAVS1), 1 ~ 2% (PITX3), 0% (OCT4)	Yes (AAVS1, OCT4), N.D. (PITX3)	TALENs	Plasmid	[51]
**HDR for Gene Correction or Modification**						
Human ESCs	Removal of Integrated GFP	0.24%	N.D.	ZFNs	Plasmid	[52]
Human ESCs	Introduction of HindIII cleavage site into EMX1	0.4%	No	Cas9 nickase mutant	Plasmid/sgRNA/Oligo	[42]
Mouse ESCs	Crytg with 1 bp deletion in exon3	0 ~ 44.4%	Yes	Cas9	Plasmid	[35]

ESCs; embryonic stem cells, NHEJ; non-homologous end-joining, HDR; homology-directed repair, GFP; green fluorescent protein, PURO; puromycin, sgRNA; single guide RNA, N.D.; not determined.

*Biallelic modification.

Such outcome suggests the attainability of genome editing-mediated gene correction in some cases of human ESCs if modified ESCs are carefully analysed regarding the occurrence of off-target mutations. Subsequently, a karyoplast removed from a genetically corrected ESC can be transferred to an enucleated oocyte retrieved from a female parent, and the resultant embryo can be cultured and transferred to the female recipient. The step is also considered to be potentially feasible in human because a similar approach with somatic cells modified by ZFNs has already been used for the generation of a biallelic knockout in pigs [61]. Additionally, human somatic cell nuclear transfer (SCNT)-derived blastocysts have been recently produced by at least three independent groups [62–64]. Remarkably, the approach does not imply cloning of a living human if this is used only once for the birth of one child. Nonetheless, the ESC approach has some disadvantages. There might be a potential risk of xeno contamination if mouse feeder cells, fetal bovine serum, or recombinant growth factors from non-human species are used in human ES medium. However, this risk can be circumvented if a xeno-free culture system is adopted. Moreover, although electroporation or transfection reagents might cause cytotoxicity [52], preliminary research would decrease such risks. Most importantly, uncertainties may occur due to the complexity of this approach. Human ESCs have a progressive tendency to acquire genetic changes in the nucleus and/or mitochondria during prolonged culture [65–68]. Moreover, not all ESC colonies in a dish are composed of the same clones. Mutant clones might be mixed into a colony during a subsequent NT procedure.

In contrast to the ESC approach, zygote approach is best characterized by fewer steps (Figure 2). Genome editing system is simply injected into the cytoplasm or pronuclear of zygotes to correct a mutation in a gene. After embryo screening, one or more embryos which have a corrected gene with no off-target mutations are then subjected to embryo transfer. Non-invasive prenatal genetic testing, which uses maternal blood containing cell-free fetal DNA [69], can be used to confirm the genetic condition of the fetus. Subsequently, invasive genetic testing, such as chorionic villus sampling or amniocentesis, can confirm whether a fetus has genetic mosaic mutations, although these diagnostics are associated with a potential risk of miscarriage. Long-term follow-up would be required even after a successful birth owing to the contribution of the modified germline to the entire body.

Microinjection of genome editing system into mammalian zygotes frequently results in efficient gene modification. According to recent reports, the efficiency of indel in a single gene by TALENs or Cas9 ranges from 0.5% to 40.9% per injected zygotes (Table 2). Remarkably, the efficiency, 40.9% was attained in non-human primate (NHP) embryos by injecting Cas9 system composed of mRNAs and sgRNAs. In this experiment, a set of twin female neonates with both modified *Rag1* and *Ppar-*γ were born. In addition, Ran *et al.* reported that Cas9 nickase treatment can induce indels in *Mecp2* at 80 to 100 percent of mouse blastocysts [42]. Regarding gene modification in neonates, the efficiencies of indel and gene addition are 0 to 41.7% by TALENs or Cas9, and 1.7 and 3.0% by Cas9, respectively (Table 2). In the targeted gene correction or modification, the efficiency is 2.0 to 6.0% in mouse neonates (Table 2). However, these gene correction or modification experiments resulted in the occurrence of off-target mutations (Table 2). The use of Cas9 nickase mutant resulted in less off-target mutations than wild type Cas9, but could not solve the off-target problem completely [44]. The Cas9-mediated HDR by an exogenously provided oligonucleotide or the endogenous wild type allele was accompanied by rare but significant off-target mutations in the mouse gene correction experiment [35]. Moreover, although no detectable mutations were found in the predefined potential off-target sites in the modified NHPs [33, 34], in another NHP experiment, a modified monkey which was treated with TALENs appeared to be mosaic [47] (Table 2).

**Table 2:** **Genome editing of mammalian zygotes**

Subject	Targeted Gene	Efficiency in Embryos ^*^	Efficiency in Neonates ^**^	Off-target Mutation	Genome Editing	Delivery Method	Remarks	Ref.
**NHEJ for InDel**								
Monkey, zygotes	Nr0b1,Ppar-γ, Rag1	18.2 ~ 40.9%, (Single), 9.1 ~ 27.3% (Double)	-	No	Cas9	mRNA/sgRNA	A set of twin female monkeys with modified Rag1 and Ppar-γ were born.	[33]
Monkey, zygotes	MECP2	-	9.5% (Rhesus), 3.7% (Cynomolgus)	No	TALENs	Plasmid	Three miscarried rhesus and cynomologus male fetuses had Mecp2 mutations.	[34]
Monkey, zygotes	MECP2	-	(2.0%)	N.D.	TALENs	mRNA	A modified male monkey appeared to be mosaic.	[47]
Bovine, zygotes	LDLR	3.8%	-	N.D.	TALENs	mRNA	Cytoplasmic injection	[46]
Porcine, zygotes	RELA	0.5%	-	N.D.	TALENs	mRNA	Cytoplasmic injection	[46]
Rat, zygotes	IgM	-	3.9 ~ 5.5% (mRNA)	No (Plasmid), Yes (mRNA)	TALENs	Plasmid or mRNA	Mosaic mutations occurred. Plasmid; Pronuclear, mRNA; Cytoplasmic injection.	[39]
Rat, Zygotes	Tet1,Tet2,Tet3	-	14.3 ~ 18.8% (Double; Tet1,2), 18.6% (Triple)	Yes (Triple)	Cas9	mRNA/sgRNA	Mosaic mutations occurred. Cytoplasmic injection	[41]
Mouse, Zygotes	Tet1,Tet2,Tet3, Sry, Uty	-	8.0 ~ 17.6% (Single), 14.7 ~ 15.3% (Double; Tet1,2)	No (Tet1,Tet2), N.D. (Tet3)	Cas9	mRNA/sgRNA	Pronuclear injection	[43]
Mouse, zygotes	Mecp2	80 ~ 100%^†^	-	No	Cas9, nickase mutant	mRNA/sgRNA	Cytoplasmic injection	[42]
Mouse, zygotes	Exo1	1.4 ~ 6.8%	0 ~ 10.3%	N.D.	TALENs	mRNA	Pronuclear injection	[48]
Mouse, zygotes	Fgf10	-	14.3 ~ 41.7%	N.D.	Cas9	mRNA/sgRNA	Cytoplasmic injection	[45]
Mouse, zygotes	Fgf10	-	1.3 ~ 1.5%	N.D.	TALENs	mRNA/sgRNA	Cytoplasmic injection	[45]
**HDR for Gene Addition**								
Mouse, zygotes	mCherry into Nanog, GFP into Oct4	-	1.7% (Nanog), 3.0% (Oct4)	Yes, (Nanog, Oct4)	Cas9	mRNA/sgRNA/Plasmid	Cytoplasmic or pronuclear injection	[40]
**HDR for Gene Correction or Modification**								
Mouse, zygotes	Addition of V5 to Sox2, lox to Mecp2	-	6.0% (Sox2), 0.8% (Mecp2)	Yes (Mecp2)	Cas9	mRNA/sgRNA/Oligo	Cytoplasmic or pronuclear injection	[40]
Mouse, zygotes	Crytg with 1 bp deletion in exon3	-	4.4 ~ 5.7%	Yes	Cas9	mRNA/sgRNA/ Oligo	Cytoplasmic injection	[35]
Mouse, zygotes	Introduction of a STOP codon into Fah	-	2.0% (wild type), 2.0% (nickase mutant)	Yes, (mutant < WT)	Cas9 WT and mutant	mRNA/sgRNA/Oligo	Pronuclear injection	[44]

*Genetically modified embryos per injected zygote (%). **Genetically modified neonates (including fetus) per transferred embryo (%). †Genetically modified blastocysts per blastocyst which underwent Cas9 treatment (%).

Zygote approach requires the PGD from the cleavage-stage (on day 3 of development) to blastocyst stage (on day 5 of development) to confirm no off-target mutations and complete correction of a mutation prior to embryo transfer. Although no mutations were detected in the predefined potential off-target sites in the NHPs in previous studies [33, 34], careful prior investigation is needed to assess whether PGD can definitely confirm genetic conditions in modified embryos. The PGD entails the opening of the zona pellucida and the removal of embryonic cell(s) from an embryo [60]. It implies that the embryo undergoes physical interventions twice, namely, microinjection of the genome editing system, and the biopsy for PGD. If ICSI is used to increase a success rate of fertilization and avoid polyspermy, three interventions are conducted. Such physical interventions might affect the subsequent development of the embryos *in vitro* or *in vivo*. Moreover, a PGD is also challenging and needs preclinical optimization because accurate genetic testing depends on biopsied embryonic cell(s). Since a cleavage-stage embryo is composed of six to eight cells, a single cell biopsy is widely used for PGD [60]. However, mosaicism which affects 15-80% of embryos may impact the interpretation of PGD results [70–72]. Meanwhile, in the blastocyst stage, the embryo consists of approximately 130 cells in the inner cell mass which subsequently develops into the fetus and the surrounding trophectoderm. Recently, trophectoderm cells have been biopsied from a blastocyst for PGD in order to avoid damaging the embryo [60]. Although mosaicism remains at the blastocyst stage [70–72], the result of a recent randomized clinical trial supports that a single cell biopsy at the cleavage-stage is more significantly damaging to the embryo than biopsy at the blastocyst stage, and resulted in poorer clinical outcomes [73]. Therefore, sufficiently optimized, trophectoderm biopsy-based PGD may be effective in the zygote approach.

Furthermore, the microinjection of genome editing system into one-cell-stage embryos needs scrutiny at the molecular level. The nuclear status transitions occur during the one-cell-stage, encompassing the separated oocyte and sperm pronuclei, pronuclear fusion, and cleavage to the two-cell-stage. Currently, pronuclear injection and cytoplasmic injection are adopted to introduce genome editing system into mammalian zygotes (Table 2). For this reason, the injection method and timing of microinjection must be optimized since incomplete gene correction by inappropriate microinjection may fail to prevent a genetic disease. In addition, the cytotoxicity caused by the genome editing system introduced in the form of a plasmid, mRNA, or protein, with or without the short repair template DNA, should be respectively investigated [21]. In doing so, the best dose should also be considered to assure a complete DNA repair with less cytotoxicity. In recent two reports, it was demonstrated that ZFN and TALEN proteins are capable of crossing cell membranes and inducing endogenous gene disruption [34, 74]. This approach, despite difficulty in the preparation of the protein, has some advantages over DNA-based delivery methods. This delivery method can limit the time that cells are exposed to such nucleases, potentially minimizing off-target activity. Moreover, this method reduces the cell-type dependency and toxicity of viral and nonviral gene delivery systems.

Collectively, the zygote approach has advantages over the ESC approach in terms of its simplicity, implying that it may be more controllable protocol. Additionally, the zygote approach is not associated with the potential ethical issues of human cloning discussed in the ESC approach. If more efficient gene correction is attained by improved genome editing, and if 13 to 15 oocytes, which is optimum number of oocytes for a successful first IVF cycle [75, 76], can be retrieved from female patients, zygote approach is more likely to be feasible in a clinical setting. One of the latest genome editing system, Cas9 is increasingly used for zygote approach due to the ease of preparation (Table 2). In contrast, Cas9 is considered to cause higher off-target mutations than ZFNs and TALENs [42]. However, Cas9 has been rapidly improved, demonstrating that the combination of a Cas9 nickase mutant and paired gRNAs, the truncation of gRNAs, or the fusion of inactive Cas9 to Fok I nuclease can improve the specificity of targeted gene modification [42, 77, 78]. Although there might be difficulties in preclinical optimization, rapid advances in genome editing would make technical obstacles surmounted, and develop germline gene correction into a medical procedure in the immediate future.

### International regulatory landscape

The preclinical research being performed to optimize the microinjection of genome editing system into one-cell-stage embryos requires human embryos for research use. However, for ethical reasons, many countries or states have strict regulations regarding the creation of human embryos for research [79]. Yet, in some countries, surplus cryopreserved embryos which were originally created by IVF or ICSI and are no longer used for reproduction are available, and researchers are permitted to derive ESCs from the surplus embryos as long as they have informed consent of the parents who underwent fertility treatment, after approval of an institutional review board (IRB) or equivalent bodies, and/or a national review. Such surplus IVF embryos might facilitate optimizing the microinjection procedure if the culture period is within the 14th day of embryo development or until the beginning of formation of primitive streak [79].

**Figure 3:**
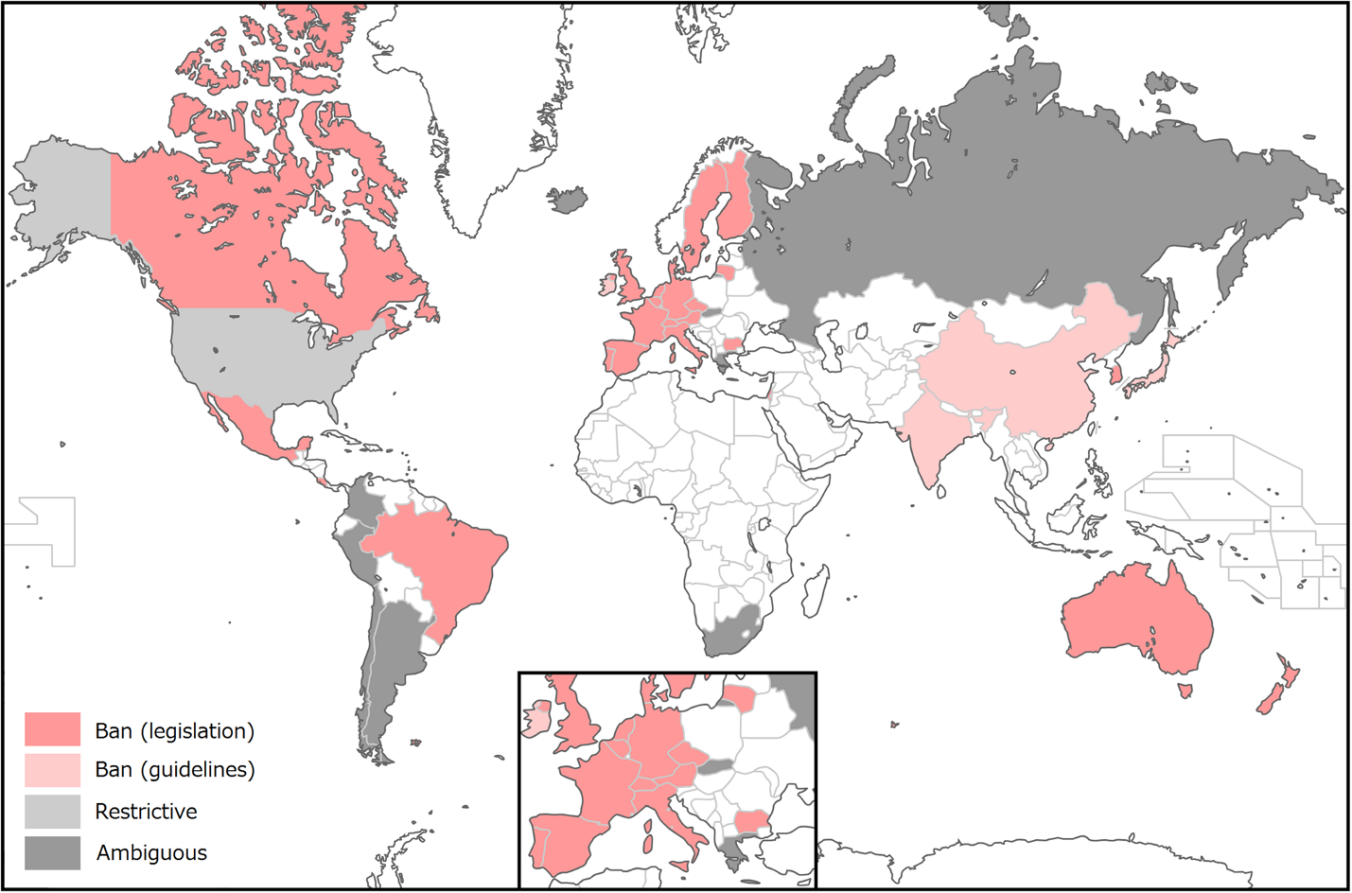
**An international regulatory landscape regarding human germline gene modification.** Thirty nine countries were surveyed and categorized as “Ban based on legislation” (25, pink), “Ban based on guidelines” (4, faint pink), “Ambiguous” (9, gray), and “Restrictive” (1, light gray). Non-colored countries were excluded in this survey. See also Additional file 1: Table S1.

More importantly, as mentioned in the Background, many countries ban human germline gene modification. We recently surveyed fourteen countries which are permissive to human ESC research, with regard to whether these countries permit human germline gene modification [9]. The result showed that thirteen of these countries prohibit the gene modification, and in the USA, FDA regulates the clinical trial, whereas the NIH restricts the application of germline gene modification. In order to examine an international regulatory landscape, we expanded our survey to 39 countries which included the 14 countries. As a result, 29 countries were found to ban germline gene modification. The remaining 10 countries include 9 countries which were ambiguous about the legal status of the modification, and the USA (Figure 3, see Additional file 1: Table S1).

Among the 29 countries, China, India, Ireland, and Japan forbid it based on guidelines that are less enforceable than laws, and are subject to amendment (Additional file 1: Table S1). The regulatory landscape suggests that human germline gene modification is not totally prohibited worldwide although there is room for further investigation regarding the “ambiguous” countries. Again, the USA currently does not ban, but has imposed a temporary moratorium on the germline gene modification under the FDA vigilance and the NIH guidelines (Additional file 1: Table S1). When the safety of genome editing-mediated germline gene correction is enhanced, the four countries mentioned above and the USA might permit it. In addition, Israel, which explicitly bans germline gene modification, but has possible exemptions in the relevant law may permit it upon the recommendation of an advisory committee [9]. This Israeli law is temporary legislation until May 23, 2016 (Additional file 1: Table S1). After that, the country might permit human germline gene modification. In the UK, the DH will consider the timing of the regulations to permit mitochondrial replacement that is currently illegal mtDNA alternation in the germline [19]. However, carefully taking into consideration that there is no legal ban on research on the human germline gene modification as long as the Human Fertilisation and Embryology Authority (HFEA) licenses such research in the UK, the legalization of medical use of mitochondrial replacement is likely to lead to legal permission for the modification of germline nuclear genome that can be readily changed by genome editing technology [80].

We recently argued that there are indistinct regulatory boundaries regarding genome editing technology created in the regulations that govern genetically modified organisms (GMOs) [81]. One of the major issues was how organisms modified using genome editing are viewed in the process-based or product-based GMO regulations. A similar debate may occur regarding the medical use of genome editing. Remarkably, Belgium, Bulgaria, Canada, Denmark, Sweden, and the Czech Republic ban germline gene modification on the grounds that a modified gene may be inherited by offspring or that the gene modification may impair human embryo (Additional file 1: Table S1). However, it is unclear whether genome editing-mediated germline gene correction is rendered illegal in those countries when genome editing can more efficiently correct a mutation in the germline. One would assert that such an act is legal, because the HDR-mediated germline gene correction results in a wild type status that can contribute to normal embryonic development. In contrast, others would dissent from this assertion, because genome editing-mediated germline gene modification can be regarded as a grave intervention in human life. Many arguments are more likely to occur with respect to the lawfulness of genome editing-mediated germline gene modification for medical purposes.

### Ethical and social issues

In an IRB review of an application for the HDR-mediated germline gene correction, the unavailability of informed consent from an unborn child may constitute grounds for ethical refusal. Yet, informed consent to the germline gene correction by parents may be justified if its safety is equivalent to that of ART, such as ICSI, which are currently conducted with informed consent provided by prospective parent(s) [9]. Still, there are difficult questions to be addressed. The condition of both parents would be questioned from various viewpoints. For instance, a board member might ask whether a female parent can safely undergo oocyte retrieval which encompasses the need of medication, a hormone injection, and transvaginal retrieval with a potential risk of ovarian hyperstimulation syndrome [82], in addition to pregnancy and delivery. Moreover, another member might ask whether the affected parents can foster the healthy child that was born of the procedure.

From a societal viewpoint, different issues would emerge when genome editing-mediated germline gene correction is practiced for preventive medicine. A regulatory agency would require that practitioners should fulfil long-term monitoring and healthcare of children born using the procedure because it could be associated with a potential risk of health impairment. However, it is difficult to determine how long such children must be monitored. Does the monitoring last during their whole lives or across several generations of the offspring? With regard to the mitochondrial replacement, the UK HFEA declared that it would be necessary to monitor the resulting children during their lifetime and ensure the traceability of gametes and embryos [83]. However, it is unlikely to be possible to perform such monitoring in all relevant countries. Moreover, it is also difficult to decide whether a country should aid all patients with thousands of genetic diseases, or how to select the subjects for the preventive medicine. If childbirth with a genetic disease no longer occurs in a country due to the extensive practice of the preventive medicine, it might impact the rights of the disabled with the genetic disease, intentionally or unintentionally assuming a posture against the existing patients who deserve respect, dignity, and support. Social cost of monitoring and healthcare would increase, as the practice of the preventive medicine grows. There is another social issue associated with healthcare costs. ART is generally expensive and creates disparities in access to this infertility services even in a country or a state with insurance coverage [84, 85]. The access to this preventive medicine would be completely confined to the wealthier segment. Thus, there are many ethical and social issues that should be addressed prior to the initiation of genome editing-mediated germline gene correction for preventive medicine.

## Conclusions

Genome editing-mediated germline gene correction for preventive medicine appears to be an unprecedented event in human history, since humans can correct a genetic mutation in the embryo using this biotechnology and potentially eradicate a congenital anomaly. We predict that corrective genome editing should reach a safe level that permits clinical applications in the immediate future. Each country will need to consider whether corrective genome editing in the human germline should be permitted with respect to socioethical implications as well as safety and efficacy. Furthermore, if a country positively considers the preventive medicine, the country would be required to express preventive measures against abuses of germline genome editing, and a global consensus will need to be formed, because thinking about germline gene modification involves ethical, social, and evolutionary considerations for all of humankind.

## Electronic supplementary material

Additional file 1: Table S1: Policies on Human Germline Gene Modification for Reproduction Excluding Reproductive Cloning. (XLSX 20 KB)
